# Assessment of the Performance of Siemens Scopio Digital Morphology on Bone Marrow Aspirates in Onco‐Hematology

**DOI:** 10.1111/ijlh.70138

**Published:** 2026-05-03

**Authors:** Gina Zini, John Marra, Elena Rossi, Silvia Bellesi, Nicoletta Pelliccioni, Giuseppe d'Onofrio, Patrizia Chiusolo

**Affiliations:** ^1^ Sezione di Ematologia, Dipartimento di Scienze Radiologiche ed Ematologiche Università Cattolica del Sacro Cuore Rome Italy; ^2^ Dipartimento di Diagnostica per Immagini, Radioterapia Oncologica ed Ematologia Fondazione Policlinico Universitario “A. Gemelli” IRCCS Rome Italy

**Keywords:** artificial intelligence, blood morphology, bone marrow aspirate, digital morphology, full‐field smear analysis, onco‐hematological disorders

## Abstract

**Objectives:**

Digital morphology (DM) systems assisted by artificial intelligence are increasingly being introduced into hematology laboratories; however, data on their performance in routine clinical practice for bone marrow aspirates (BMA) remain limited. We evaluated the automated pre‐classification generated by a full‐field DM system in a large series of BMA samples from patients with onco‐hematological disorders.

**Methods:**

We analyzed 350 BMA samples during routine diagnostic activity; they were evaluated using the Siemens Scopio X100 HT system. Automated results were compared with conventional optical microscopy (OM), which served as the reference method for bone marrow differential counts. Results obtained in pre‐classification and post‐classification were analyzed separately across major cellular lineages and clinically relevant blast thresholds.

**Results:**

Sixteen markedly hypercellular samples could not be quantitatively evaluated. In the 334 evaluable BMA samples, the granulocytic series showed very strong correlation between DM and OM at pre‐classification (*r* = 0.85, 95% CI 0.82–0.88), with further improvement after post‐classification (*r* = 0.93, 95% CI 0.91–0.94). Erythroblast percentage showed strong correlation at pre‐classification (*r* = 0.78, 95% CI 0.74–0.82) and very strong correlation after post‐classification (*r* = 0.93, 95% CI 0.92–0.95). Lymphocyte percentage showed moderate correlation at pre‐classification (*r* = 0.55, 95% CI 0.47–0.62) and strong correlation after post‐classification (*r* = 0.78, 95% CI 0.73–0.82). Blast percentage showed strong correlation overall at pre‐classification (*r* = 0.73, 95% CI 0.67–0.77), with very strong correlation in samples with < 5% blasts (*r* = 0.91, 95% CI 0.88–0.93), but lower correlation in samples with > 5% blasts (*r* = 0.59, 95% CI 0.48–0.68), improving after post‐classification (*r* = 0.84, 95% CI 0.78–0.88). Low‐frequency cell populations and challenging hypercellular smears remained more problematic and required expert morphologic review.

**Conclusions:**

In routine BMA evaluation, the Scopio DM system provided good overall performance for the major marrow lineages, with clear improvement after expert post‐classification. Its main value lies in supporting a supervised diagnostic workflow rather than replacing expert microscopic assessment, particularly in challenging samples and in low‐frequency or morphologically heterogeneous cell populations.

## 1 Introduction

The diagnosis of hematologic diseases relies on an integrated assessment combining clinical features with morphologic examination, flow cytometric immunophenotyping, and molecular testing. Current classification systems, including WHO HAEM5 and the International Consensus Classification [1, 2], highlight the central role of genomic information in defining disease entities and guiding prognostic evaluation. Concurrently, diagnostic laboratories are facing increasing case complexity alongside limited human resources. To address these challenges, advanced technologies such as laboratory automation, collaborative robotics, digital morphology (DM) platforms, and artificial intelligence (AI) are progressively being implemented. DM has significantly advanced hematologic diagnostics by providing new methods to streamline and standardize the analysis of peripheral blood smears (PB) [3] Automated systems are becoming more common in daily laboratory routines for PB cell morphologic pre‐classification, and the literature assesses their performance under real‐world conditions by comparing automated results with conventional OM, which remains the gold standard [4, 5, 6]. The introduction of DM systems for bone marrow aspirate (BMA) smear pre‐classification represents an important opportunity to enhance the diagnostic process by harmonizing and standardizing workflows and reducing turnaround time (TAT) [7, 8, 9, 10].

The Hematology Laboratory at Policlinico Universitario A. Gemelli‐IRCSS in Rome (Italy), Joint Commission‐500‐accredited, is open 24 h a day. Hematologists on duty annually report, according to laboratory standard operating procedures (SOPs) and international guidelines [11], at least 500 000 PB and 3300 BMA.

In February 2025, a non‐profit study was approved by the ethics committee to evaluate the clinical performance of the Scopio‐Siemens Technology applied to our routine diagnostic activity for BMA samples reporting. This study aims to evaluate the analytical performance, diagnostic reliability, and consistency of the “Scopio X100 HT Cell Morphology Analyzer IVD, automated BM and PB application” by comparing its automated results with those of the reference method, the OM, in onco‐hematological BMA samples.

## 2 Material and Methods

### 2.1 Scopio X100 HT

The Scopio X100 HT device reconstructs the full monolayer digitized area, including the “feathered edge,” to produce a continuous, high‐definition field of view. The operator can navigate the digitized slide at any time, enabling a simulated conventional optical microscopy (OM) review experience. The system integrates a high‐precision scanning module with AI algorithms that automatically identify, classify, and enumerate distinct blood cell populations, thereby enabling early detection of hematologic abnormalities [12]. Moreover, it displays sample identification data on the “Full Field Review” page, allows navigation across the entire scanned area, and reports the number of nucleated cells counted (distinguishing intact from disrupted cells), as well as the number of megakaryocytes and detected fragments. A macroscopic image of the entire slide is also provided, clearly outlining the scanned region. The scanning time varies from 15 to 30 min for the BM slide and 7 min for the PB slide. If necessary, scanning can be repeated by manually selecting a different area of the smear.

The “Cell Review” page provides images of individual cells grouped into the following categories: (i) Myeloid lineage (blast, promyelocyte, myelocyte, metamyelocyte, band, and neutrophil), (ii) Lymphoid lineage (plasma cells and lymphocytes), (iii) Erythroid lineage (proerythroblast, basophilic, polychromatophilic and orthochromatic erythroblast), (iv) Monocyte, Eosinophil, Basophil, Mast cell, and Unclassified, reported as separate categories. The total myelogram is expressed both as an absolute count and as a percentage for each category. The Scopio default cell enumeration setting was 500, in agreement with the ICSH guidelines for BMA [11].

The proposed myeloid‐to‐erythroid (M:E) ratio is calculated from the ratio between elements included in the myeloid lineage and those in the erythroid lineage. In addition to the myelogram, absolute counts are provided for macrophages, other damaged cells, artifacts, and megakaryocytes.

Individual cells can be directly selected from the whole‐slide image with a single click, with magnification up to 200%. Cells may be reclassified by moving them from one category to another, and the system automatically recalculates the differential count. All manual modifications are traceable. In selected patients at diagnosis, PB analysis was performed using the Atellica HEMA 580 Analyzer system, connected to the Atellica HEMA Slidemaker stainer for PB smear preparation and automatic slide staining, and the Scopio System then analyzed the slides for digital pre‐classification.

### 2.2 Sampling

This observational study includes 350 BMA samples collected during routine diagnostic activity from patients with onco‐hematological disorders at diagnosis, during follow‐up, or at relapse. For automated BMA scanning, we used the same manually prepared, automatically stained slides used for official reporting. According to our SOPs, BMA were manually wedge‐spread and automatically stained using the May‐Grünwald‐Giemsa method with a Delcon Aerospray Hematology Pro slide stainer.

### 2.3 Study Design

Our aim was to assess the performance of automated pre‐classification by the Scopio DM system in a large series of BMA samples analyzed in routine clinical practice, by comparing its results with those of the reference method.

Conventional OM served as the reference method for BM differential counts. In accordance with our certified SOPs, each BM report was independently reviewed by two expert hematologists, with a third senior reviewer involved in cases of discordance.

Semi‐quantitative parameters (particle density, cellularity, and megakaryocyte assessment) were standardized and aligned with DM quantitative outputs in accordance with institutional reporting standards.

The aggregation of selected data was based on clinical relevance and reporting efficiency, in accordance with our internal SOPs for BMA evaluation.

Quantitative data were correlated as follows: (i) overall percentages for the erythroid and neutrophilic granulocytic series were reported by merging the respective intralineage subcategories; (ii) lymphocytes, plasma cells, basophils, eosinophils, monocytic series, mast cell lineages were correlated separately; (iii) agranular blasts, granular blasts, monoblasts, lymphoblasts, morphologically unidentifiable blasts, blast equivalents, immature lymphoma cells, and plasmablasts were grouped and correlated with the Scopio blast category.

The myeloid‐to‐erythroid (M:E) ratio generated by the system was excluded from the evaluation because it is calculated using criteria that differ from those of the reference guidelines [11]. Specifically, the system includes lymphoblasts, when present, among blasts and excludes basophils, eosinophils, monocytes, and mast cells from the ratio calculation.

### 2.4 Statistics

Correlation between DM and OM quantitative results was assessed using Pearson's correlation coefficient (*r*). Corresponding 95% confidence intervals (CIs) were calculated by the Fisher *z* transformation. Each analysis included only cases with complete paired numeric data. Correlation strength was interpreted descriptively according to the magnitude of *r* as very weak (0.00–0.19), weak (0.20–0.39), moderate (0.40–0.59), strong (0.60–0.79), and very strong (0.80–1.00). Results are presented as *r* with 95% CI (rounded to two significant digits).

Three analytical stages were assessed:

1. Automated results without human intervention (pre‐classification).
2. Results after expert post‐classification, when necessary.
3. Discrepancy analysis: all discrepancies were individually analyzed to determine their causes. Clinically significant discrepancies between DM and OM were re‐evaluated by repeat OM review of the corresponding BMA smears.

## 3 Results

### 3.1 Sample Characteristics and Evaluability

Among 350 scanned samples, 334 yielded quantitative differential results. Sixteen samples (< 5%) from patients with leukemia and leukemic lymphomas had dense, thick, markedly hypercellular smears and yielded only slide images without reliable differential output. The distribution of diagnoses and clinical phases of 334 evaluated samples is summarized in Table 1.

**TABLE 1:** Diagnosis and clinical phase of 334 evaluated bone marrow samples.

Diagnosis		Clinical phase				
		First observation	Relapse	Follow‐up	Post‐alloBMT	Post‐CAR‐T
Myelodysplastic neoplasms	11% (38)	26	0	12	0	0
Myeloproliferative neoplasms	4% (13)	9	2	2	0	0
Myelodysplastic/Myeloproliferative neoplasms	2% (7)	4	1	1	0	1
Acute myeloid leukemia^a^	43% (142)	76	2	57	5	2
Precursors B‐ and T‐cell neoplasms	10% (35)	24	2	5	3	1
Mature B‐, T‐ and NK‐cell neoplasms/B and T Lymphomas, multiple myelomas	25% (84)	40	2	37	4	1
Miscellaneous (hypoplasia/aplasia, PNH, ITP, unexplained Cytopenia(s), and reactive bone marrow)^b^	5% (15)	10	0	5	0	0
Total	100%	189	9	119	12	5

^a^ Includes 2 cases of biphenotypic acute leukemia and 1 case of blastic plasmacytoid dendritic cell neoplasm.

^b^ PNH: paroxysmal nocturnal hemoglobinuria; ITP: immune thrombocytopenic purpura.

### 3.2 Semi‐Quantitative Data From the BMA

Table 2 summarizes the alignment between OM semi‐quantitative and DM quantitative outputs. In the 334 evaluable samples, the number of nucleated cells ranged from 200 to 500 by OM and from 56 to 1901 by DM. Particle evaluation was available in 295 samples (88%), and megakaryocyte assessment in 285 samples (85%).

**TABLE 2:** Parametric framework: Semi‐quantitative vs. quantitative data for BM particles and megakaryocytes.

Number of samples	Optical microscope cell count: 200–500	Scopio digital morphology cell count: 56–1901
	OM semi‐quantitative particle estimation	DM quantitative—particles: 295/334 samples (88%)
4	Absent	0
18	Rare	≥ 1 ≤ 3
16	Present	> 3 ≤ 5
185	Several	> 6 ≤ 10
72	Numerous	> 10

	Megakaryocytes OM range	Quantitative—Megakaryocytes: 285/334 (85%) (postvalidation)
3	Apparently absent	0
32	Apparently absent	1–7
58	Rare	1–16
145	Present/Normal	4–34
47	Numerous/highly increased	15–36

### 3.3 Granulocytic Lineage

**FIGURE 1:**
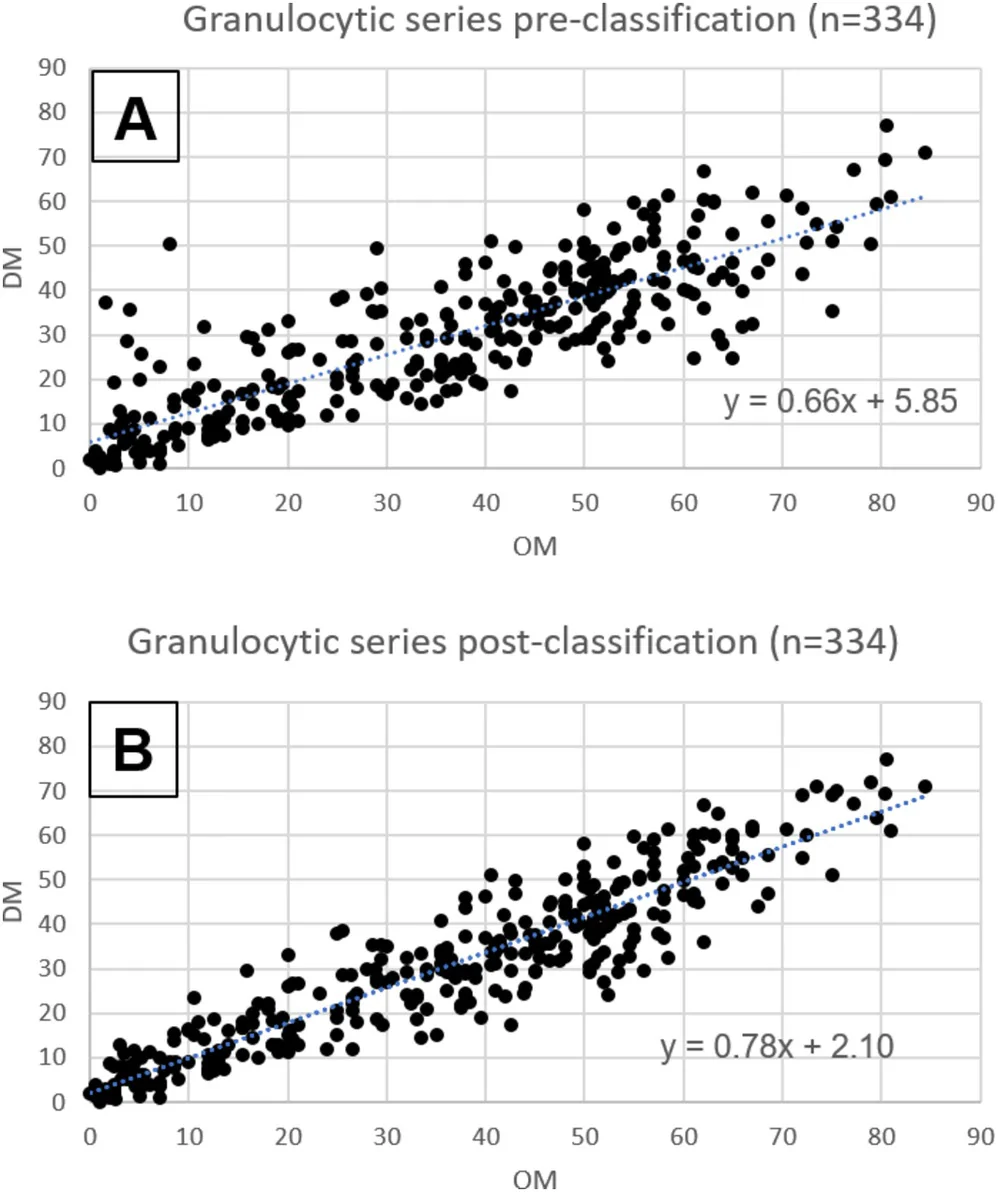
Granulocytic series. Correlation between optical microscope (OM) and Scopio digital morphology (DM) pre‐classification (A) and post‐classification (B).

In the overall cohort of 334 samples, the granulocytic series showed a very strong correlation between DM and OM at pre‐classification (*r* = 0.85, 95% CI 0.82–0.88) and an even stronger correlation after post‐classification (*r* = 0.93, 95% CI 0.91–0.94). In a subset analysis of 314 cases, excluding 20 highly discrepant APL and AML samples with monocytic lineage involvement, pre‐classification correlation remained very strong (*r* = 0.90, 95% CI 0.87–0.92) (Figure 1A–B).

### 3.4 Erythroid Lineage

In the overall cohort of 334 samples, correlation between DM and OM erythroblast percentage was strong at pre‐classification (*r* = 0.78, 95% CI 0.74–0.82) and became very strong after post‐classification (*r* = 0.93, 95% CI 0.92–0.95). Misclassification occurred mainly in samples containing small‐sized blasts, lymphoma cells or plasma cell (Figure 3A,B).

### 3.5 Lymphocytes

In the entire cohort of 334 samples, correlation between DM and OM lymphocyte percentage was moderate at pre‐classification (*r* = 0.55, 95% CI 0.47–0.62) and improved to strong after post‐classification (*r* = 0.78, 95% CI 0.73–0.82). In samples with different types of lymphoma cells, many cells were pre‐classified as the “unclassified” category. By contrast, in 15 samples of chronic lymphocytic leukemia, characterized by moderate to high infiltration of small, monomorphic, mature lymphocytes, the clonal population was largely pre‐classified as lymphocytes.

### 3.6 Blast Count

**FIGURE 2:**
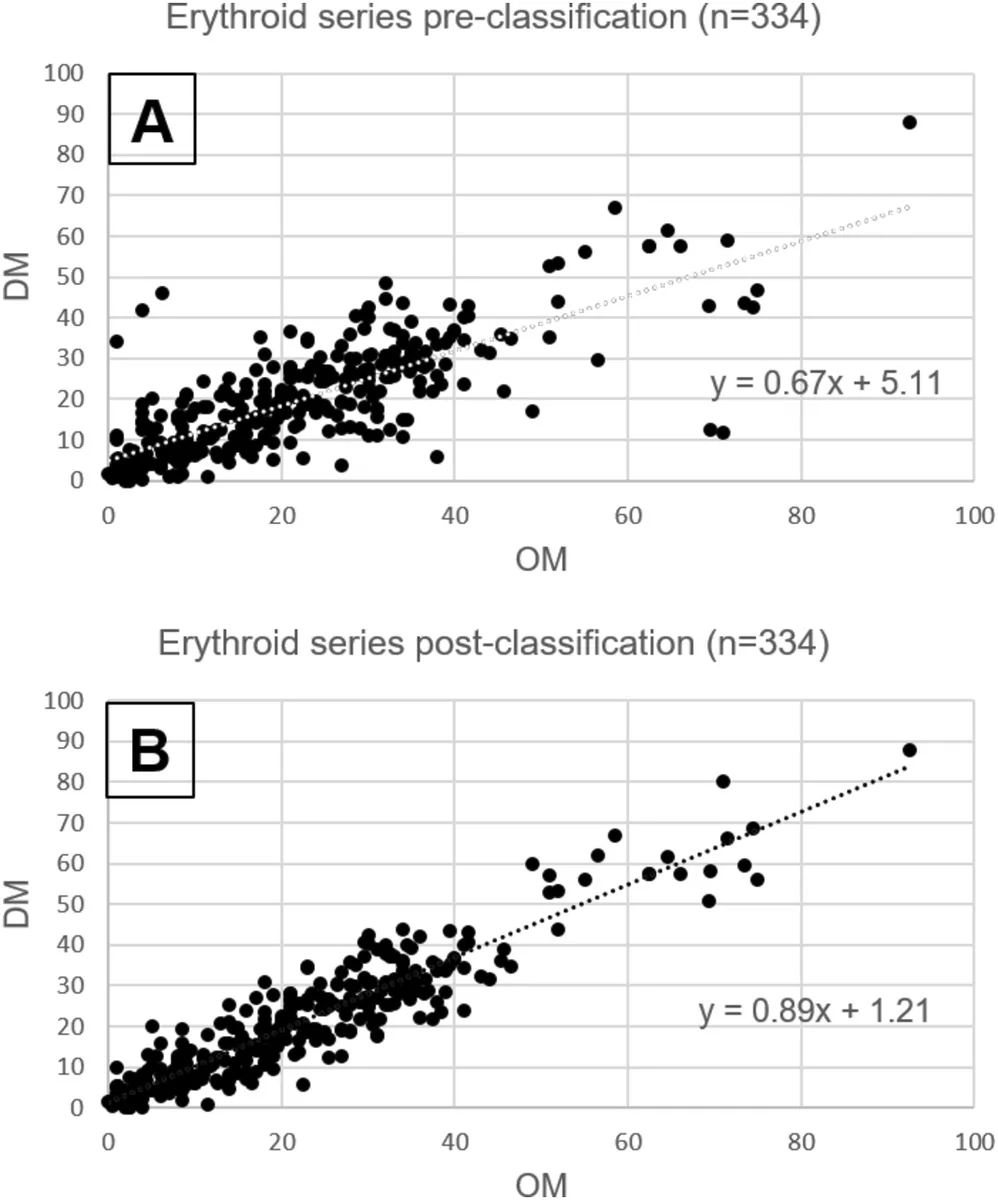
Erythroid series. Correlation between optical microscope (OM) and Scopio digital morphology (DM) preclassification (A) and postclassification (B).

In the overall cohort of 334 samples, blast percentage showed a strong correlation between DM and OM at pre‐classification (*r* = 0.73, 95% CI 0.67–0.77). Thirteen acute leukemia samples were not appropriately evaluable in the post‐classification stage because of insufficient image quality of the abnormal cells, due to overcrowded smears, focusing issues, or abnormal cell distribution with densely packed areas. In the cohort of 321 in which the on screen post‐classification was carried out, the blast percentage showed a very strong correlation between DM and OM at post‐classification (*r* = 0.97, 95% CI 0.96–0.98). In the 174 samples with < 5% blasts, pre‐classification already showed very strong correlation (*r* = 0.91, 95% CI 0.88–0.93). In the 147 samples with > 5% blasts, correlation was moderate at pre‐classification (*r* = 0.59, 95% CI 0.48–0.68) and improved to very strong after post‐classification (*r* = 0.84, 95% CI 0.78–0.88). Seven samples diagnosed with acute promyelocytic leukemia, including five cases of hypergranular and two cases of microgranular variants, remained morphologically recognizable on screen during post‐classification. These results are shown in Figure 2A–C.

### 3.7 Megakaryocytes

**FIGURE 3:**
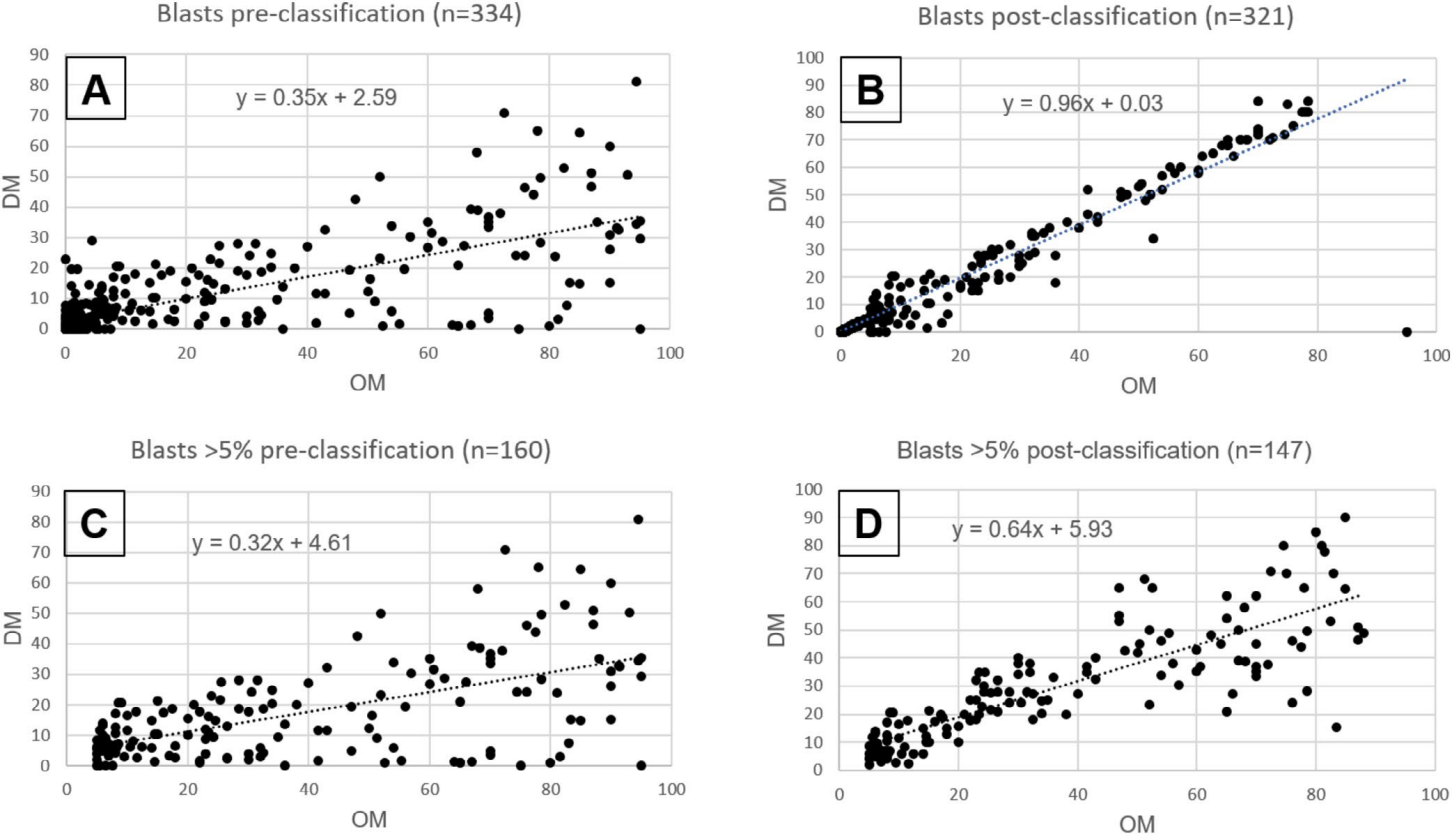
Blast cells. Correlation between optical microscope (OM) and Scopio digital morphology (DM). (A) Pre‐classification of all samples; (B) Postclassification of all evaluable samples; (C) Preclassification of samples with > 5% blasts; (D) Post‐classification of samples with > 5% blasts.

Megakaryocyte assessment was particularly informative in hypocellular samples. In cases in which OM reported an apparent absence of megakaryocytes, the digital system was often still able to identify isolated megakaryocytes within the scanned smear area. Reviewing a broader smear surface and examining large cells at high resolution was especially helpful when megakaryocytes were sparse and irregularly distributed (Figure 3).

### 3.8 Low‐Frequency Cell Populations

Low‐frequency cell populations were more difficult to assess by automated pre‐classification than the major marrow lineages, mainly because of their rarity, morphologic heterogeneity, and uneven distribution on the smear. Nevertheless, the full‐field digital image and the ability to review individual cells in their morphologic context remained useful. Mature plasma cells were usually recognizable on screen, whereas immature or atypical forms more often required post‐classification. Eosinophils were generally well identified, although eosinophilic precursors were occasionally assigned to more mature categories. The monocytic lineage was more challenging, particularly in samples with clonal expansion, where distinction among mature monocytes, immature forms, promonocytes, and monoblasts was not always satisfactory at pre‐classification. Mast cells and basophils, because of their low frequency and atypical appearance, were also prone to misclassification.

### 3.9 Dysplastic Features

In several samples with evident dyserythropoietic changes, dysplastic erythroblasts remained morphologically recognizable on screen. Dysplastic granulocytic and megakaryocytic features were likewise evident in all evaluable samples.

## 4 Discussion

The present study evaluated the performance of an AI–assisted DM system for automated pre‐classification of BMA under routine diagnostic conditions in a large tertiary‐care hematology laboratory. Unlike controlled validation studies, the cohort included a broad spectrum of onco‐hematological diseases, clinical situations, and smear qualities, reflecting everyday diagnostic activity.

Automated pre‐classification provided a reasonable approximation of the differential count for the major cellular lineages, particularly granulocytic and erythroid elements, whereas performance was more variable for blasts and lymphoid populations. The image resolution of cellular details, together with the ability to review individual categorized cells within the full‐field scan, allowed more reliable morphologic reassessment during the post‐classification phase in evaluable samples. As for APL, the high reproducibility of morphological details on screen during post‐classification, such as granules, Auer rods, chromatin patterns, and nuclear shapes, enabled reliable recognition of diagnostically relevant cytological features in this disease, which is considered high‐risk if not diagnosed promptly. Notably, even in samples with marked dyserythropoiesis, the system preserved morphologic features allowing dysplastic erythroblasts to remain recognizable as erythroid cells. The high reproducibility of cellular details on post‐classification enables accurate detection of dysplasia in digital BM reporting [13]. As for the low‐frequency cell population, they were better handled in a supervised review setting than by automated pre‐classification alone, and their diagnostic assessment remained primarily dependent on expert morphologic interpretation.

A consistent observation was the clear improvement observed after expert post‐classification. This indicates that current DM platforms are better suited as support tools than as autonomous diagnostic systems.

Agreement with OM was highest in samples with low blast burden. In cases with < 5% blasts, automated estimates were close to the reference values, suggesting that the system can reliably recognize normal or near‐normal marrow patterns. This point is clinically relevant, since many bone marrow examinations are performed during follow‐up rather than at initial diagnosis [14]. Accuracy decreased in samples with higher blast percentages, where cellular heterogeneity, atypical morphology, and crowding effects are more prominent. Distinguishing immature cells from abnormal precursors on morphology alone is known to be difficult [14, 15], even for experienced observers, and this difficulty is reflected in the automated results.

The findings in acute promyelocytic leukemia illustrate both the limitations and the usefulness of the digital approach. During automated pre‐classification, abnormal promyelocytes were often distributed across different categories, especially in the microgranular variant. After expert review, however, the image resolution enabled reliable recognition of diagnostic features, including cytoplasmic granules, nuclear morphology, and Auer rods. The main advantage of the system, therefore, lies less in unsupervised classification than in the possibility of rapid reassessment within a comprehensive digital environment.

DM appeared to provide useful support for samples with low megakaryocyte counts: in our cohort, in 32 cases where the reference method reported megakaryocytes as “apparently absent,” the system clearly identified megakaryocytes and reported them within a range of 1–7 cells. Performance for lymphoid populations was lower than for myeloid or erythroid elements, particularly in cases with clonal infiltration. Many atypical lymphoid cells were assigned to the “unclassified” category, likely reflecting their limited representation in current algorithm training datasets. From a diagnostic standpoint, this limitation may be less critical than suggested by numerical discrepancies, because abnormal lymphoid infiltrates are usually recognized on qualitative morphological grounds. The ability to examine these cells at high resolution after classification remains clinically useful [16].

Low‐frequency cell types, such as mast cells, basophils, and immature plasma cells, showed poor agreement with automated analysis compared with OM. These populations are rare and morphologically heterogeneous, and even manual quantification is subject to variability. In most cases, however, these cells were identifiable during expert review, indicating that the system does not prevent recognition but may not quantify them accurately without supervision.

A small proportion of samples could not be quantitatively evaluated because of extreme hypercellularity, uneven cell distribution, or technical artifacts. Similar conditions complicate conventional microscopy, although experienced observers can often compensate by examining selected areas of the smear. The automated system's inability to handle such cases underscores the importance of smear quality for digital analysis and confirms that pre‐analytical factors remain critical [17, 18, 19, 20, 21].

DM also offers practical advantages that are not captured by correlation statistics alone [7, 8, 9, 10, 13]. Full‐field scanning allows review of the entire smear without physical slide manipulation, facilitates remote consultation, and provides permanent image storage. These characteristics may support quality control, education and training activities [16, 17], enable rapid second opinions, and improve workflow organization, particularly in laboratories with high diagnostic workload.

Scanning time efficiency allows rapid identification of unexpected pathological samples during routine workflow, facilitating prompt diagnostic management. In particular, cytological observation and discussion of dyserytropoietic features are enhanced by on screen visualization, possibly reducing interobserver variation [18].

From an operational perspective, the distinction between automated pre‐classification and expert post‐classification provides a realistic model for routine implementation. Automated results can serve as an initial screening tool, identifying abnormal samples and providing a general overview, while final interpretation remains under human control [22, 23, 24, 25]. Such an approach may reduce observer fatigue and optimize time management without affecting diagnostic reliability.

Our study has several limitations. It was conducted in a single tertiary‐care center with experienced morphologists and a specific case mix. The reference method, although based on certified SOPs (verificare) and double review, remained based on morphology, which makes it subject to intrinsic observer variability (subjectivity). Some clinically relevant subgroups, including rare cell populations and markedly hypercellular samples, were underrepresented or not fully analyzable. In addition, our statistical analysis was based primarily on method correlation, and the findings may not be directly generalizable to laboratories using different smear preparation procedures, staining protocols, or software versions. In summary, automated DM provides useful preliminary information for bone marrow aspirate evaluation but does not replace expert microscopic assessment. Its main contribution lies in supporting a human‐supervised diagnostic process, especially in high‐volume laboratories where efficiency and reproducibility are important considerations. Further studies will be needed to define the long‐term clinical impact, cost‐effectiveness, and possible applications in training and telehematology.

## Funding

The study was funded by Fondazione Policlinico Gemelli and Siemens Scopio Labs.

## Ethics Statement

The study was approved by the CET (Territorial Ethical Committee) Lazio Area 3, ID 6901, on July 25, 2024, in accordance with the Helsinki Declaration.

## Consent

The authors have nothing to report.

## Conflicts of Interest

Gina Zini: Honoraria as speaker from Mindray and Siemens Corporations. The other authors declare no conflicts of interest.

## Data Availability

Data generated in this study are available from the authors upon reasonable request and with permission of Scopio Labs.

## References

[1] D. A. Arber , The International Consensus Classification of Myeloid and Lymphoid Neoplasms First Edition (Wolters Kluwer Editor ISBN, 2025).

[2] 5th WHO classification of tumors series , 5th ed. Hematolymphoid tumors (International Agency for Research on Cancer, 2022), 10.1038/s41375-023-01962-5.

[3] A. CSH Kratz , S. Lee , G. Zini , et al., “Digital Morphology Analyzers in Hematology: ICSH Review and Recommendations,” International Journal of Laboratory Hematology 41 (2019): 437–447. 31046197 10.1111/ijlh.13042

[4] G. Da Rin , M. Seghezzi , A. Padoan , et al., “Multicentric Evaluation of the Variability of Digital Morphology Performances Also Respect to the Reference Methods by Optical Microscopy,” International Journal of Laboratory Hematology 44 (2022): 1040–1049. 35916349 10.1111/ijlh.13943

[5] V. De Iuliis and R. S. Chiatamone , “Performance Evaluation of the Scopio Labs X100HT Digital Morphology Analyzer and Abnormal Cell Detection in Peripheral Blood Smears,” International Journal of Laboratory Hematology 48, no. 1 (February 2026): 54–62, 10.1111/ijlh.70005. 40947947

[6] B. Z. Katz , D. Benisty , Y. Sayegh , I. Lamm , and I. Avivi , “Remote Digital Microscopy Improves Hematology Laboratory Workflow by Reducing Peripheral Blood Smear Analysis Turnaround Time,” Applied Clinical Informatics 13 (2022): 1108–1115. 36208622 10.1055/a-1957-6219 PMC9683999

[7] A. Bagg , P. W. Raess , D. Rund , et al., “Performance Evaluation of a Novel Artificial Intelligence‐Assisted Digital Microscopy System for the Routine Analysis of Bone Marrow Aspirates,” Modern Pathology 37, no. 9 (September 2024): 100542. 38897451 10.1016/j.modpat.2024.100542

[8] X. Fu , M. Fu , Q. Li , et al., “Morphogo: An Automatic Bone Marrow Cell Classification System on Digital Images Analyzed by Artificial Intelligence,” Acta Cytologica 64 (2020): 588–596. 32721953 10.1159/000509524

[9] J. E. Lewis , C. W. Shebelut , B. R. Drumheller , et al., “An Automated Pipeline for Differential Cell Counts on Whole Slide Bone Marrow Aspirate Smears,” Modern Pathology 36 (2023): 100003. 36853796 10.1016/j.modpat.2022.100003 PMC10310355

[10] C. W. Wang , S.‐C. Huang , Y.‐C. Lee , Y.‐J. Shen , S.‐I. Meng , and J. L. Gaol , “Deep Learning for Bone Marrow Cell Detection and Classification on Whole‐Slide Images,” Medical Image Analysis 75 (2022): 102270. 34710655 10.1016/j.media.2021.102270

[11] S. H. Lee , W. N. Erber , A. Porwit , M. Tomonaga , and L. C. Peterson , “International Council for Standardization in Hematology. ICSH Guidelines for the Standardization of Bone Marrow Specimens and Reports,” International Journal of Laboratory Hematology 30 (2008): 349–364. 18822060 10.1111/j.1751-553X.2008.01100.x

[12] B. Z. Katz , M. D. Feldman , M. Tessema , et al., “Evaluation of Scopio Labs X100 Full Field PBS: The First High‐Resolution Full Field Viewing of Peripheral Blood Specimens Combined With Artificial Intelligence‐Based Morphological Analysis,” International Journal of Laboratory Hematology 43, no. 6 (December 2021): 1408–1416. 34546630 10.1111/ijlh.13681 PMC9293172

[13] P. Font , J. Loscertales , C. Soto , et al., “Interobserver Variance in Myelodysplastic Syndromes With Less Than 5% Bone Marrow Blasts: Unilineage vs. Multilineage Dysplasia and Reproducibility of the Threshold of 2% Blasts,” Annals of Hematology 94 (2015): 565–573. 25387664 10.1007/s00277-014-2252-4

[14] K. R. Calvo , A. Dulau , I. Maric , J. Sun , and R. Braylan , “The Challenging Task of Enumerating Blasts in the Bone Marrow,” Seminars in Hematology 56 (2019): 58–64. 30573046 10.1053/j.seminhematol.2018.07.001

[15] K. Foucar , E. D. Hsi , S. A. Wang , et al., “Bone Marrow Pathology Group. Concordance Among Hematopathologists in Classifying Blasts Plus Promonocytes: A Bone Marrow Pathology Group Study,” International Journal of Laboratory Hematology 42 (2020): 418–422. 32297416 10.1111/ijlh.13212

[16] A. Carobene , J. Cadamuro , G. Frans , et al., “EFLM Checklist for the Assessment of AI/ML Studies in Laboratory Medicine: Enhancing General Medical AI Frameworks for Laboratory‐Specific Applications,” Clinical Chemistry and Laboratory Medicine 64, no. 1 (2025): 27–40. 40966119 10.1515/cclm-2025-0841

[17] H. Kim , M. Hur , G. d'Onofrio , and G. Zini , “Real‐World Application of Digital Morphology Analyzers: Practical Issues and Challenges in Clinical Laboratories,” Diagnostics (Basel) 15, no. 6 (2025): 677. 40150020 10.3390/diagnostics15060677 PMC11941716

[18] G. Zini , P. Chiusolo , E. Rossi , et al., “Digital Morphology Compared to the Optical Microscope: A Validation Study on Reporting Bone Marrow Aspirates,” International Journal of Laboratory Hematology 46, no. 3 (2024): 474–480, 10.1111/ijlh.14238. 38328984

[19] J. E. Lewis and O. Pozdnyakova , “Advances in Bone Marrow Evaluation,” Clinics in Laboratory Medicine 44, no. 3 (2024): 431–440. 39089749 10.1016/j.cll.2024.04.005

[20] G. Zini , “Hematological Cytomorphology: Where We Are,” International Journal of Laboratory Hematology 46, no. 5 (2024): 789–794. 38898733 10.1111/ijlh.14330

[21] S. Sun , Z. Yin , J. G. Van Cleave , et al., “DeepHeme, a High‐Performance, Generalizable Deep Ensemble for Bone Marrow Morphometry and Hematologic Diagnosis,” Science Translational Medicine 17, no. 802 (2025): eadq2162, 10.1126/scitranslmed.adq2162. 40498857

[22] G. Zini , Y. H. Chang , G. d'Onofrio , et al., “ICSH Recommendations for Monocyte Cell Lineage Morphologic Identification, Nomenclature Harmonization, and Utilization as a Biomarker,” International Journal of Laboratory Hematology 48, no. 1 (2026): 12–25. 41297912 10.1111/ijlh.70029 PMC12809381

[23] G. Zini , O. Barbagallo , F. Scavone , and M. C. Béné , “Digital Morphology in Hematology Diagnosis and Education: The Experience of the European LeukemiaNet WP10,” International Journal of Laboratory Hematology 44, no. Suppl 1 (2022): 37–44. 36074713 10.1111/ijlh.13908

[24] J. Burthem , M. Brereton , J. Ardern , et al., “The Use of Digital ‘virtual Slides’ in the Quality Assessment of Haematological Morphology: Results of a Pilot Exercise Involving UKNEQAS(H) Participants,” British Journal of Haematology 130 (2005): 293–296. 16029459 10.1111/j.1365-2141.2005.05597.x

[25] K. Naqvi , E. Jabbour , C. Bueso‐Ramos , et al., “Implications of Discrepancy in Morphologic Diagnosis of Myelodysplastic Syndrome Between Referral and Tertiary Care Centers,” Blood 118 (2011): 4690–4693. 21868570 10.1182/blood-2011-03-342642 PMC4081364

